# Supplementary material for: Factor Structure and Measurement Invariance of the Very Short Form of Infant Behavior Questionnaire-Revised (IBQR-VSF): A Study among Vietnamese Children
Source: Healthcare (Basel). 2022 Apr 6;10(4):689. doi: 10.3390/healthcare10040689 (PMC9031564; doi:10.3390/healthcare10040689)
Supplement: Supplementary file 1 [file healthcare-10-00689-s001.zip › healthcare-1625303-supplementary.pdf]

Supplementary

Table S1. Items excluded due to the low Chronbach's α value.

**Surgency**	
Item 7	How often during the week did your baby move quickly toward new objects?
Item 15:	How often does the infant look up from playing when the telephone rings?
Item 20:	When visiting a new place, how often did your baby get excited about exploring new surroundings?
Item 21	How often during the last week did the baby smile or laugh when given a toy?
Item 27	How often did your baby notice the sound of an airplane passing overhead?
**Negative affect**	
Item 3:	When tired, how often did your baby show distress?
Item 17:	How often during the last week did the baby startle at a sudden change in body position (e.g., when moved suddenly)?
Item 23	How often during the last week did the baby protest being placed in a confining place(infant seat, play pen, car seat, etc.)?
**Orienting regulation**	
Item 11	In the last week, while being fed in your lap, how often did the baby seem eager to get away as soon as the feeding was over?
